# Bioresorbable scaffolds vs. drug-eluting stents on short- and mid-term target lesion outcomes in patients after PCI: A systematic review and meta-analysis

**DOI:** 10.3389/fcvm.2022.949494

**Published:** 2022-09-08

**Authors:** Yan-di Wan, Da-yang Wang, Wen-qi Deng, Si-jia Lai, Xian Wang

**Affiliations:** ^1^Dongzhimen Hospital, Beijing University of Chinese Medicine, Beijing, China; ^2^Institute of Cardiology, Beijing University of Chinese Medicine, Beijing, China

**Keywords:** bioresorbable scaffolds, drug-eluting stents, systematic review, meta-analysis, outcomes

## Abstract

**Background:**

While current concerns about bioresorbable scaffolds (BRS) are centered on late or very late scaffold thrombosis, less attention had been paid to short- and mid-term clinical outcomes. This review aimed to compare the short- and mid-term outcomes between BRS and drug-eluting stents (DES).

**Methods:**

A systematic review of randomized controlled trials (RCTs) that compared BRS vs. DES was conducted by searching PubMed, Cochrane Library, Web of Science, CNKI, WanFang, and VIP databases from inception until 19 April 2022 (language limited to English or Chinese). The primary outcome was target lesion failure (TLF) within 12 months, defined as a composite of target lesion revascularization (TLR), target vessel myocardial infarction (TVMI), and cardiac death. The secondary outcomes were in-stent diameter stenosis (DS%) provided by intraluminal imaging.

**Results:**

A total of 13 studies were eligible and were included in this review (*N* = 9,702 patients). The follow-up duration ranged from 6 months to 1 year. A significantly higher rate of TLF [RR, 1.22, 95% CI (1.03, 1.44)] driven by the higher rate of TVMI [RR, 1.39, 95% CI (1.09, 1.76)] was observed in the BRS group than in the DES group. The risk of TLR and cardiac death was similar between the groups. Also, compared with the DES group, the BRS group had a significantly higher in-stent DS% within 1 year [MD = 5.23, 95%CI (3.43, 7.04); I^2^ = 97%; *p* < 0.00001].

**Conclusion:**

Bioresorbable scaffolds were associated with an increased risk of target lesion failure within 1 year as compared with DES, driven by the increased rates of target vessel myocardial infarction. Also, the in-stent DS% seemed to be higher with BRS. Therefore, BRS was inferior to DES in terms of target lesion outcomes at short- or mid-term follow-up.

**Systematic review registration:**

https://www.crd.york.ac.uk/PROSPERO/display_record.php?RecordID=327966, PROSPERO (CRD42022327966).

## Introduction

Since the time drug-eluting stents (DES) were discovered, many trials and studies have shown their superiority to bare-metal stents (1). In addition, among DES, second-generation devices have substantially improved long-term safety and efficacy outcomes compared with first-generation devices (1, 2). Therefore, second-generation DES have been widely recommended for PCI (3). Nevertheless, studies also showed that DES was linked to neoatherosclerosis and fracture-related adverse pathological events (4, 5). Due to the presence of a permanent metallic stent, numerous deleterious long-term effects may occur, including vessel straightening, loss of compliance, vasoregulation, adaptive remodeling, and the potential for late inflammation and mechanical failure (6, 7). Therefore, the concept of bioresorbable scaffolds emerged. BRS was designed with the aim of reducing long-term adverse events stemming from permanent materials (8). To date, multiple trials evaluating clinical outcomes with BRS have been performed, including observational, non-comparative/single-arm studies, and large-scale random controlled trials (9, 10). Based on those studies, many prior meta-analyses focusing on long-term efficacy and safety (i.e., beyond 1 year) suggested that BRS was associated with worse clinical outcomes and a higher risk of adverse events, including device thrombosis, compared with DES (9, 11–14). However, much less emphasis has been placed on short- and mid-term clinical outcomes of BRS. Although several meta-analyses showed that BRS was inferior to DES at 1-year follow-up, these reviews did not include all the randomized controlled trials (RCTs) so far (15, 16). As more results of large-scale RCTs have been presented in recent years, we aimed to conduct a meta-analysis involving all the RCTs up to now. Moreover, compared with other previous reviews, this review emphasized more in terms of neointimal hyperplasia and lumen loss.

## Methods

### Data sources and searches

This review was performed according to the Preferred Reporting Items for Systematic Reviews and Meta-Analyses Statement 2020 (17). We systematically searched PubMed, Cochrane Library, Web of Science, CNKI, WanFang database, and VIP databases from inception until 19 April 2022. The language was limited to Chinese or English. References of the included studies were also searched manually to supplement relevant articles. Searches were conducted using a combination of subject terms and free words. English keywords included “bioresorbable vascular stent,” “target lesion failure,” and “randomized controlled trial.” See Appendix 1 in the Supplementary material for search strategies in detail.

### Eligibility criteria

Studies were considered eligible for inclusion if they were RCTs performed in patients undergoing PCI with both a clinical and an imaging diagnosis of CAD (including SCAD and ACS). Patients were randomized to treatment with either BRS or DES, regardless of the brands, types, lengths, and material parameters of stents and scaffolds. Duplicate publications were excluded (e.g., publications using the same data in different languages or partial data from a large-scale study).

### Outcomes and definitions

The setting of composite endpoints for this review was based on the standardized definitions from the Academic Research Consortium (ARC) (18). Our primary outcome was target lesion failure (TLF), defined as target lesion revascularization (TLR), target vessel myocardial infarction (TVMI), and cardiac death. The RCTs included should provide at least one of the outcomes mentioned above at 6–12 months of follow-up after PCI. The secondary outcome was in-stent diameter stenosis (in-stent DS%). Studies enrolled should provide in-stent DS% as reported by coronary angiography, IVUS, or optical coherence tomography (OCT) at 6 to 12-month follow-up after PCI.

### Study selection and data extraction

Searches and study selection were conducted independently by two reviewers (Yan-di Wan and Da-yang Wang). The process advanced as follows: (I) read study titles and exclude the studies not related to bioresorbable scaffolds, (II) read the abstracts and exclude non-RCTs (cohort comparison studies and nested case-control studies), (III) read the original text and exclude the studies that did not report the outcomes mentioned in the Outcomes section, and (IV) further read the full text and exclude duplicate publications. Finally, the results of searches and selections were cross-checked. Any discrepancy regarding searches and selection was discussed in consultation with and resolved by a third reviewer (Wen-qi Deng).

After identifying the included studies, two reviewers independently conducted data extraction. The data included the following: (I) basic information: study name, year of publication, country of study, sample size, and eligibility criteria; (II) baseline information: gender, age, follow-up visit, material of stent, and brand of stent; (III) outcome indicators: TLF, TLR, TVMI, cardiac death, and in-stent DS%; and (IV) assessment on the risk of bias: randomization, blinding, and allocation concealment. Data were checked after the extraction; Discrepancy, if any, was verified and resolved by a third reviewer (Wen-qi Deng). Literature was managed with the Endnote X9 software (19).

In this review, the management of missing values followed the processing method reported in the original studies. Last observation carried forward (LOCF) was a very common approach employed in trials to impute values for missing outcomes (20).

### Effect measures

Statistical analysis was performed using Revman5.4. Data extracted were entered in Revman5.4 (21). Meanwhile, the mean ± standard deviation (mean ± SD) was adopted as the effect analysis statistic for measures, and the risk ratio (RR) with corresponding 95% confidence intervals (CIs) was adopted as the effect analysis statistic for binary variables.

### Quality assessment

The assessment of study quality was performed based on the Cochrane Collaboration's tool for assessing the risk of bias (22). Each entry from the tool was adjusted according to the review. To assess the risk of bias in the included studies, the tool was adjusted based on the seven criteria, namely, random sequence generation, allocation concealment, blinding of participants and personnel, blinding of outcome assessment, incomplete outcome data, selective reporting, and other biases. The points mentioned above stood for selection, performance, detection, attrition, reporting, and other biases, respectively. According to the extracted data, each study included was rated as “low risk of bias,” “unclear risk of bias,” or “high risk of bias” based on those seven points correspondingly. Summary graphs of the risk of bias were also generated with Revman5.4 based on the rating of the studies in terms of those points.

The quality of evidence was also evaluated according to the GRADE (Grading of Recommendations Assessment, Development, and Evaluation) system. Based on the type of studies, the GRADE system graded four levels of quality (high, moderate, low, and very low). RCTs started with a high rating, and the rating was modified downward in the following situations: (1) study limitations, (2) imprecision, (3) inconsistency of results, (4) indirectness of evidence, and (5) publication bias likely. Finally, the quality of each outcome was rated as high, moderate, low, or very low (23).

### Statistical analysis

Between-study heterogeneity was evaluated using the χ^2^ test. Meanwhile, the heterogeneity was evaluated quantitatively with the Higgins I^2^ index (24). Evaluation for risk of publication bias was performed using Revman5.4, with funnel plots being generated. Also, the risk of publication bias was evaluated quantitatively using Stata 12.0 (25).

If there was no statistical between-study heterogeneity, a meta-analysis was performed with the fixed-effects model. If statistical heterogeneity existed, the source of heterogeneity was further analyzed. Then, the random-effects model was adopted after the effect of significant clinical heterogeneity was excluded (α = 0.05 was adopted as the test level of meta-analysis). The summary RRs were constructed using the DerSimonian–Laird random-effects model (26). Significant clinical heterogeneity was coped using subgroup analysis and sensitivity analysis, or descriptive analysis alone.

In this review, subgroup analysis was based on the brand of the stents in order to determine how this factor influenced the short- and mid-term outcomes of patients with CAD treated with BRS. When there was high between-study heterogeneity, each of the included studies in this review was excluded one after another in order to analyze the sensitivity of the overall results.

## Results

### Included studies and characteristics

**Figure 1:**
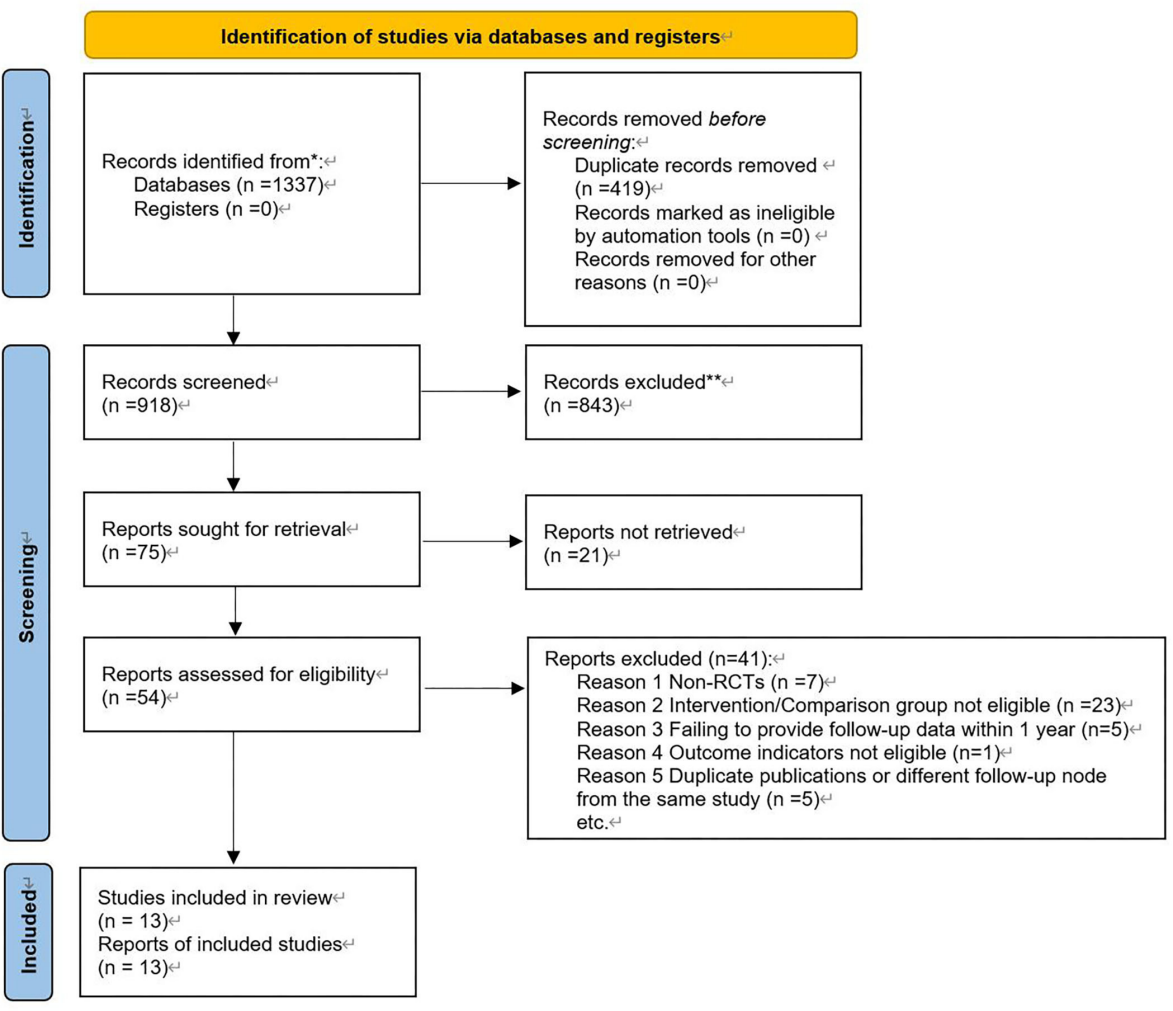
Selection process.

The electronic search yielded 1,337 articles. According to the eligibility criteria, a final number of 13 trials with 9,702 patients (with 5,328 patients in the BRS arm and 4,374 patients in the DES arm) were included in this meta-analysis (see Figure 1 for the specific selection process). All the trials were multicenter, except for the EVERBIO II trial, which was a single-center trial (27). Both the TROFI II trial and the ISAR-Absorb MI trial reported outcomes at 6 months (28, 29), while the EVEROBIO II trial reported outcomes at 9 months (27). The other 10 trials all reported outcomes at 1 year (10, 30–38). Among all the included trials, the BRS could be divided into four brands, including ABSORB BVS, NEOVAS, XINSORB, and MAGMARIS. The former three brands were PLLA-based scaffolds, and the latter one was magnesium-based. The follow-up duration reported in the studies ranged from 6 months to 1 year. The mean age of participants was 57.4–67.0 years. The percentage of women was 10.6–32.7% in those studies. The proportion of participants diagnosed with diabetes was 16.0–35.35%. Six included studies applied the “adequate pre-dilation, accurate sizing, and adequate post-dilation” (PSP) technique to PCI (refer to Table 1 for summarized baseline characteristics of the studies included).

**Table 1:** Characteristics of included studies.

**References**	**Year**	**Country**	**Inclusion and exclusion criteria**	**Reported follow-up, month**	**Patients, n**	**BRS type**	**DES type**	**Female, %**	**Age, y, mean**	**DM, %**	**Applying PSP technique**	**Remark**
ABSORB II (33)	2015	Europe and New Zealand	Normal^#^	12	501 (335/166)	ABSORB BVS™	XIENCE™	22%	61.2	24%	No	
ABORB III (30)	2015	America and Australia	Normal	12	2,008 (1,322/686)	ABSORB BVS™	XIENCE™	29.6%	63.6	32.1%	No	
ABSORB IV (10)	2018	America, Germany, Australia, Singapore and Canada	Long lesions alone were excluded	12	2,604 (1,296/1,308)	ABSORB BVS™	XIENCE™	28.1%	62.65	31.75%	Yes	
ABSORB China (31)	2015	China	Normal	12	480 (241/239)	ABSORB BVS™	XIENCE™	27.8%	57.4	24.4%	Yes	
ABSORB Japan (32)	2015	Japan	Normal	12	400 (266/134)	ABSORB BVS™	XIENCE™	23.5%	67	36%	Yes	
TROFI II (28)	2016	Europe	Only AMI was included	6	191 (95/96)	ABSORB BVS™	XIENCE™	17.9%	58.7	16.8%	No	
EVEROBIO II (27)	2015	Switzerland	Only target lesions with oversize-diameter lumen were excluded	9	189 (61/128)	ABSORB BVS™	Promus element™ or Biomatrix Flex™	21%	65	23%	No	
Neovas RCT (34)	2018	China	Normal	12	560 (277/283)	NEOVAS™	CoCr-EES	32.2%	58.7	19.5%	No	
Xinsorb RCT (36)	2019	China	Normal	12	395 (200/195)	XINSORB™	TIVOLI™	32.7%	60.1	23.0%	No	
ISAR-Absorb MI (29)	2019	Germany, Spain, Denmark, Russia	Only AMI was included	6	213 (140/73)	ABSORB BVS™	EES	23.6%	62.5	20.5%	No	
MAGSTEMI (35) (Magnesium-Based Resorbable Scaffold)	2019	Spain	Only STEMI was included	12	150 (76/74)	MAGMARIS™^*^	Orsiro™	10.6%	59.0	16.0%	Yes	
Seo et al. (37)	2020	Korea	Only long lesions >28 mm were included	12	341 (171/170)	ABSORB BVS	XIENCE™	21.7%	62.5	31%	No	
Compare ABSORB (38)	2020	Europe	Lesions with high risk of ISR were included	12	1,670 (848/822)	ABSORB BVS	XIENCE™	22.1%	62.1	35.35%	Yes	Early termination

* All the BRSs were PLLA-based except for MAGMARIS scaffolds;

# Trials without special inclusion or exclusion criteria were marked as ‘normal'. Patients with the following circumstances were excluded in trials with normal eligibility and exclusion criteria: EF <30%, renal insufficiency, high bleeding risk, AMI, left main lesions, ostial lesions, long lesions, severely tortuous lesions, bifurcation lesions, CTO lesions, small/large-diameter target vessels, myocardial bridges or other complex lesions.

### Qualitative review

**Figure 2:**
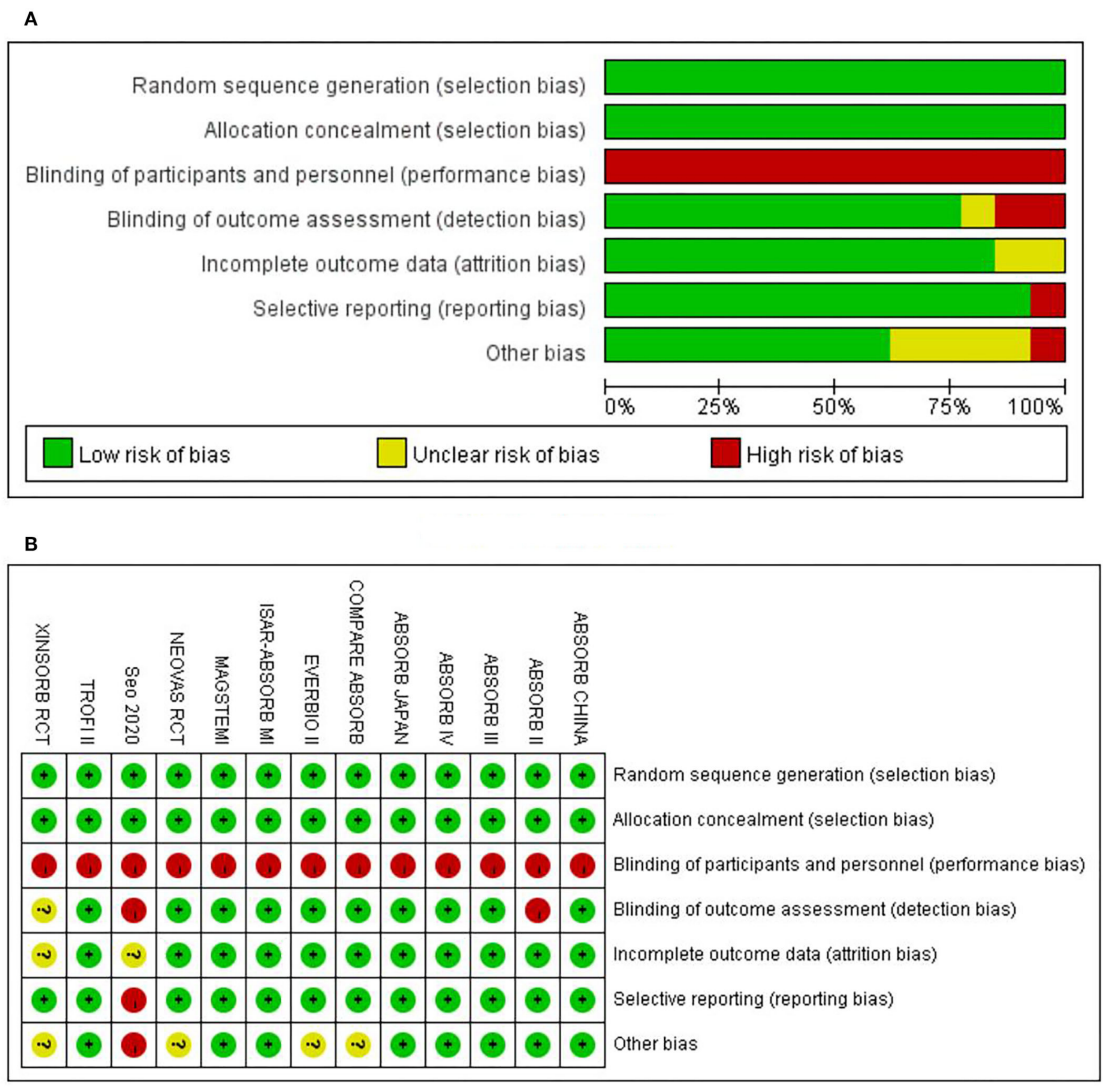
Assessment on risk of bias. **(A)** The overall risk of bias. **(B)** Risk of bias for specific studies.

**Figure 3:**
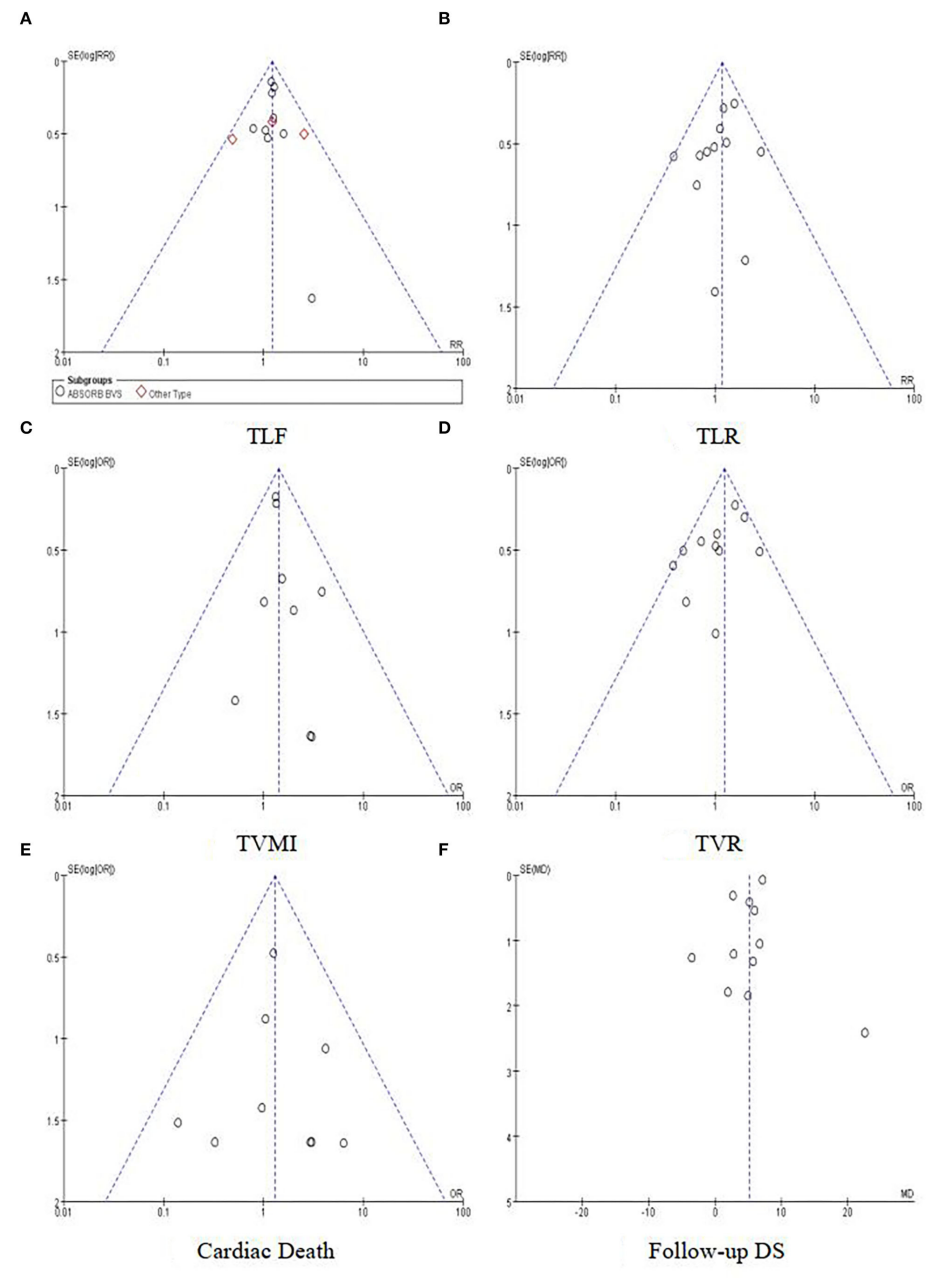
Funnel plots for outcomes. **(A)** TLF. **(B)** TLR. **(C)** TVMI. **(D)** TVR. **(E)** Cardiac death. **(F)** Follow-up DS.

The risk of bias for each included study was evaluated using the Cochrane Collaboration's tool for assessing the risk of bias. The results are shown in Figure 2. Out of the 13 included trials, 10 were rated as low risk of bias, while 2 were found to have a high risk of bias and the rest 1 was unclear in terms of risk of bias. The overall risk of bias was low. Funnel plots (Figure 3) were generated using STATA to make a qualitative analysis for publication bias. According to those figures, no significant publication bias was found. Also, Egger's test (39) and Begg's test were performed using STATA. The results showed *p* > 0.05 for each outcome indicator. Therefore, publication bias was not found.

### Outcomes

#### Primary outcome

**Figure 4:**
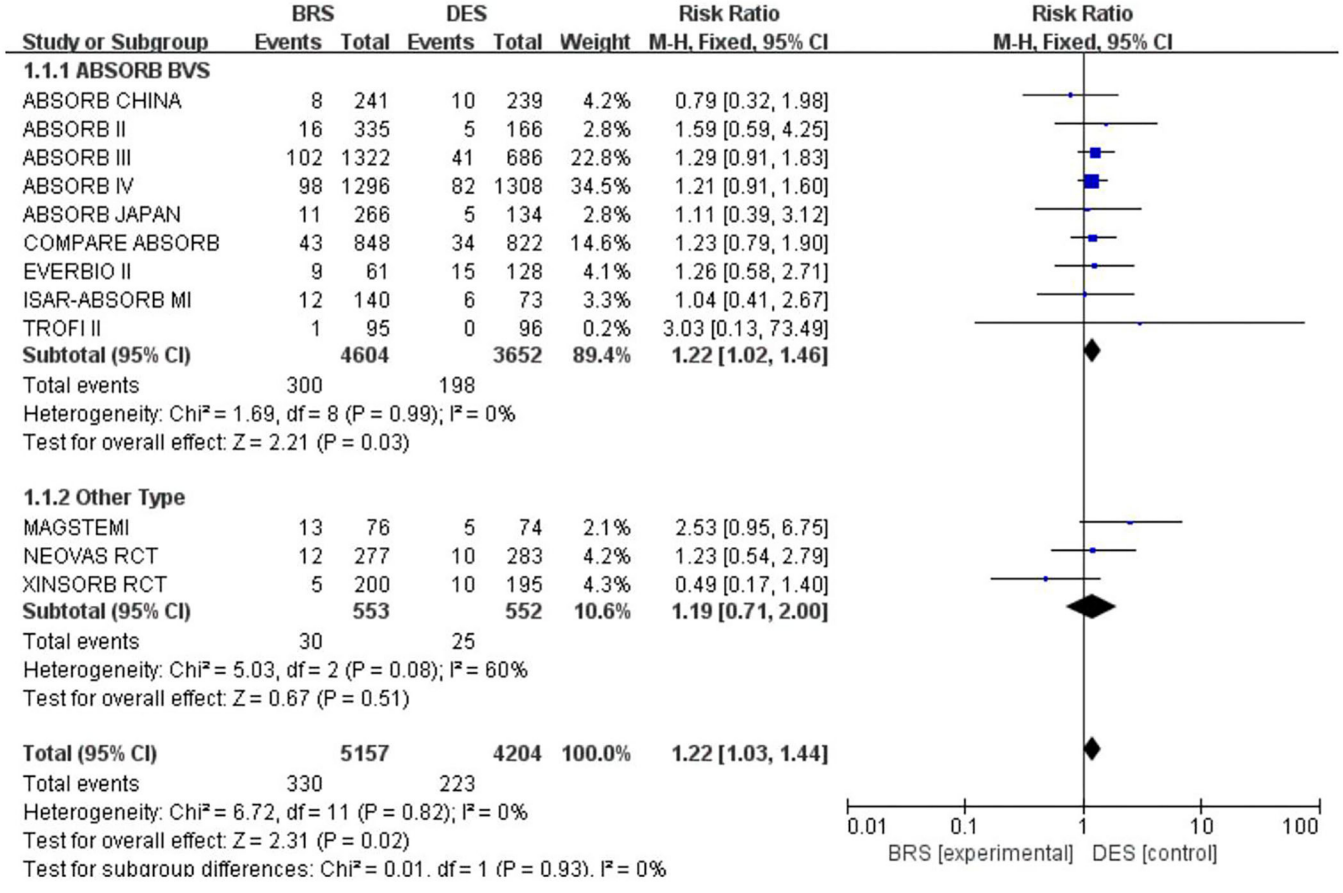
Meta-analysis results of forest plots for TLF.

A total of 12 included studies reported the TLF rate within 1 year, involving 9,361 participants. Out of 5,157 participants treated with BRS, 330 turned out to have TLF, while 223 had TLF among the 4,204 participants in the DES group. The results of the heterogeneity test showed *p* = 0.93 and *I*^2^ = 0; thus, the fixed-effects model was adopted. According to the results of the meta-analysis, the incidence of TLF in the BRS group was greater than that in the DES group [RR = 1.22, 95% CI (1.03, 1.44); *I*^2^ = 0%; *p* = 0.02], mainly driven by the higher rate of TVMI [RR, 1.39, 95% CI (1.09, 1.76); *I*^2^ = 0%; *p* = 0.008] (see Figure 4 for forest plots). Also, a subgroup analysis was additionally conducted among the studies that applied ABSORB BVS. Still, the results showed that the incidence of TLF was greater in the BRS group, with RR = 1.22, 95% CI (1.02, 1.46); *I*^2^ = 0%; *p* = 0.03. Since the heterogeneity was high among the studies applying other brands of stents, further subgroup analysis was not performed.

#### Secondary outcome

**Figure 5:**
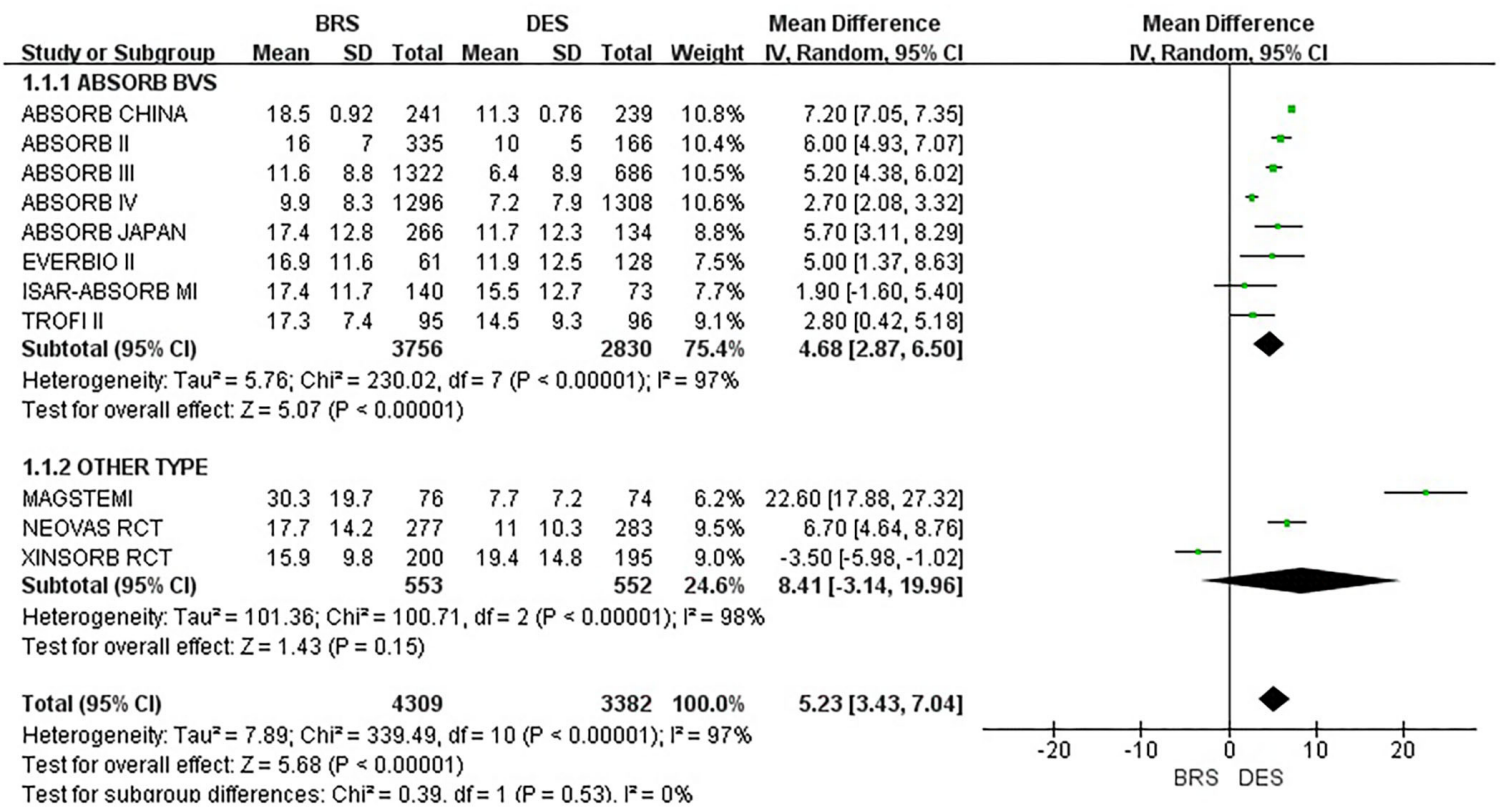
Meta-analysis results of forest plots for in-stent DS%.

A total of 11 included studies reported in-stent DS% within 1 year. The total between-study heterogeneity was high (*I*^2^ = 97%). A subgroup analysis was additionally conducted among the studies that applied ABSORB BVS and other types, respectively. However, the results showed high heterogeneity. We also performed subgroup analyses according to the sample size and AMI population, but they showed similar results. Because there were no more available data of in-stent DS% on detailed subgroups such as poststent dilation, ACS, and DM population, we were unable to further analyze the reasons for such high heterogeneity. Therefore, a random effect model was used (refer to Figure 5 for details). Compared with the DES group, the BRS group had a significantly higher in-stent DS% within 1 year [MD = 5.23, 95%CI (3.43, 7.04); *I*^2^ = 97%; *p* < 0.00001]. A sensitivity analysis was also conducted. We removed one study at a time and recalculated the combined estimate on the remaining studies. The sensitivity analysis did not identify the apparent difference.

#### Other outcomes

Other outcomes in this review included TLR and cardiac death. Compared with DES, BRS had a similar risk of TLR [RR = 1.19, 95% CI (0.92, 1.54); *I*^2^ = 0%; *p* = 0.18] and cardiac death [RR = 1.31, 95% CI (0.72, 2.38); *I*^2^ = 0%; *p* = 0.37]. The results of the meta-analysis on those outcomes are summarized in Table 2.

**Table 2:** Meta-analysis results on other outcomes.

**Outcomes**	**Heterogeneity (I^2^)**	**RR (95%CI)**	***P***
TLR	0%	1.19 (0.92, 1.54)	0.18
TVMI	0%	1.39 (1.09, 1.76)	0.008
TVR	38%	1.23 (0.98, 1.54)	0.08
Cardiac death	0%	1.31 (0.72, 2.38)	0.37

## Discussion

In this meta-analysis, a total of 13 RCTs were included, involving 9,702 patients with coronary artery disease undergoing PCI. The result indicated a higher target lesion failure in BRS compared with DES, mainly driven by a higher risk of TVMI. Also, in the BRS group, a significantly increased risk for in-stent DS% within 1 year was observed as compared to DES. The incidence of TLR and cardiac death was not significantly different between the two groups.

### Setting of outcomes

This review mainly focused on the outcomes of BRS implantation at short- and mid-term follow-up. While the current concerns about BRS centered on late/very late scaffold thrombosis (40) and late lumen loss (41), less attention had been paid to short- and mid-term follow-up. Since the peak of DS rate after stenting showed within 1 year, the incidence of TLF within 1 year was defined as the short- and mid-term outcome in this review. Generally speaking, the peak rate of neointimal hyperplasia occurs in 3–6 months (42) after BMS implantation, whereas in the case of DES, the peak rate was delayed (43) due to the application of anti-proliferative drugs. No related data on BRS were available. However, since its drug release rate is similar to that of DES, we suppose that the peak time of neointimal hyperplasia in the case of BRS may be similar to that of DES. Therefore, the follow-up was set at 6 months to 1 year in this review.

Most clinical research on coronary stents tended to adopt TLF as the primary outcome. TLF included all the events related to the target lesion directly, namely, TLR, TVMI, and cardiac death (33). They were also referred to as device-oriented composite endpoint (DoCE) in that those events reflected the safety associated with device implantation. In addition to TLF, lumen gain or loss within the stented segment was another important outcome, as it provided a more visualized comparison of the safety among different stents. In general, it was obtained through coronary angiography, OCT, or IVUS at follow-up. In this review, it was adopted as another important outcome.

### Explanation for the results

Results of the meta-analysis in this review showed a significantly greater incidence of TLF within 1 year for BRS as compared with that for DES. This was in line with the hypothesis of this review that BRS was inferior to DES in terms of improvements in short- and mid-term prognosis. Significant statistical differences were not found in any of the included studies alone (shown in Figure 4). Hence, this result could not be observed without a meta-analysis.

When analyzing the reasons for the increased risk of TVMI, we found that there was also a higher risk of definite or probable scaffold thrombosis for BRS [RR = 2.11, 95% CI (1.36, 3.28); *I*^2^ = 0%; *p* = 0.0009]. Prior data from meta-analyses and real-world studies had already shown a trend of a higher risk of definite or probable scaffold thrombosis with BRS (44–46), including early scaffold thrombosis and late/very late scaffold thrombosis (9, 11, 12), which contributed to the potentially higher risk of TLR, TVMI, and cardiac death. This was in line with the results of this meta-analysis.

Further analyzing the inferiority of BRS to DES, we found that the potential reasons may lie in three aspects, including thicker scaffold struts, poorer radial support and ductility, and the stimuli to tunica intima caused by degradation products of coating. Currently, a variety of potential matrices for BRS have been explored. Moreover, many *in vitro* and animal experiments have been completed (47) for them. However, the majority of BRS that had passed the clinical tests in humans were made of PLLA. Although PLLA had a good balance of degradation, radial support, and biocompatibility, it could not rival metallic materials. For instance, to provide sufficient radial support, the PLLA scaffold struts needed to reach a thickness of ~150 μm (34). Consequently, the scaffold strut area was nearly four times as big as a metal one after implantation, resulting in greater thrombosis risk as demonstrated in this review and other similar studies (48). Even though the thickness of the scaffold struts for BRS had reached 150 μm, BRS was still inferior to DES in terms of support and ductility. Therefore, the prognosis may be affected by stent malapposition and under-expansion, which occurred afterward. In addition to that, the acid environment created by the degradation products of PLLA itself also stimulated local inflammatory responses in the tunica intima. In brief, all the potential factors mentioned above may contribute to PLLA-based BRS being inferior to metal-based DES.

### Limitations

As far as we know, this is the largest meta-analysis of RCTs up till now evaluating the short- and mid-term outcomes of BRS compared with DES. Although this meta-analysis was conducted with a robust methodology, it was not without limitations. As is known to cardiologists and clinical researchers, stenting is a special way of intervention in which the blinding of operators is almost impossible. Additionally, a certain degree of bias may occur during the implementation of intervention because the incidence of restenosis and scaffold thrombosis is closely related to the implantation technique. As regards the outcome assessors, although they were mentioned to be blinded in most included studies, the effect of blinding on them was limited in actual evaluation. The reason may lie in the fact that compared with DES, BRS had thicker stent struts and they were not visible under radiation, which made it easy for assessors to distinguish them through angiography, OCT, and IVUS. Thus, there was a certain degree of detection bias in the assessment of stenosis degree within the stent. However, most of the studies included in this review were sponsored by BRS manufacturers. In other words, the bias may be directional. Consequently, the result of BRS leading to poor prognosis at short- and mid-term follow-up may be weakened. According to the results from this review, the adverse effect of BRS on short- and mid-term prognosis may have been underestimated. In terms of publication bias, the funnel plots and Egger's and Begg's tests have been performed and no publication bias was found.

## Conclusion

Bioresorbable scaffolds, compared with DES, had an increased risk of TLF, which was mainly driven by the higher incidence of TVMI. Also, in-stent DS% was higher with BRS. Therefore, BRS was inferior to DES at short- or mid-term follow-up in terms of target lesion outcomes. Further data of short-term follow-up from randomized trials are needed to fully evaluate and analyze the clinical outcomes with BRS before resorption of the scaffold.

## Data availability statement

The original contributions presented in the study are included in the article/Supplementary material, further inquiries can be directed to the corresponding author/s.

## Author contributions

Y-dW and D-yW drafted the initial manuscript, registered the protocol at PROSPERO, and conducted the search, study selection, data extraction, and risk of bias assessment. D-yW developed the search strategy and rechecked and analyzed the outcomes. W-qD and S-jL were responsible for checking references. All authors contributed to the review drafting and approved the final manuscript.

## Funding

The research was supported by the National Natural Science Foundation of China (81774058).

## Conflict of interest

The authors declare that the research was conducted in the absence of any commercial or financial relationships that could be construed as a potential conflict of interest.

## Publisher's note

All claims expressed in this article are solely those of the authors and do not necessarily represent those of their affiliated organizations, or those of the publisher, the editors and the reviewers. Any product that may be evaluated in this article, or claim that may be made by its manufacturer, is not guaranteed or endorsed by the publisher.
