# Supplementary material for: Bioresorbable scaffolds vs. drug-eluting stents on short- and mid-term target lesion outcomes in patients after PCI: A systematic review and meta-analysis
Source: Front Cardiovasc Med. 2022 Sep 8;9:949494. doi: 10.3389/fcvm.2022.949494 (PMC9492944; doi:10.3389/fcvm.2022.949494)
Supplement: Supplementary file 1 [file Data_Sheet_1.docx]

Appendix 1：Search Strategies

1 Bioresorbable Scaffolds versus Drug-eluting Stents on Short and Mid-term Target Lesion Outcome in Patients after PCI: A Systematic Review and Meta-Analysis.

## Pubmed (n=116)

#1：Bioresorbable[Title/Abstract]

#2：Bioabsorbable[Title/Abstract]

#3：#1 OR #2

#4：Stent[Title]

#5: Scaffold[Title]

#6: Stents[title]

#7: Scaffolds[title]

#8: #4 OR #5 OR #6 OR #7

#9：Target Lesion Failure[Title/Abstract]

#11: Revascularization[Title/Abstract]

#12：Lumen Loss[Title/Abstract]

#13: LL[Title/Abstract]

#14: In-segment Late Loss[Title/Abstract]

#15：Restenosis[Title/Abstract]

#16：Cardiac Death[Title/Abstract]

#17：Target Vessel Myocardial Infraction[Title/Abstract]

#18: Adverse Events[Title/Abstract]

#19: Endpoint[Title/Abstract]

#20: Endpoints[Title/Abstract]

#21：#9 OR #10 OR #11 OR #12 OR #13 OR #14 OR #15 OR #16 OR #17 OR #18 OR #19 OR #20

#22: Human[Filter] OR Randomized Controlled Trial[Filter]

#23：#3 AND #8 AND #21 AND #22

(Bioresorbable[Title/Abstract] OR Bioabsorbable[Title/Abstract]) AND (Stent[Title] OR Scaffold[Title] OR Stents[title] OR Scaffolds[title]) AND (Target Lesion Failure[Title/Abstract] OR Revascularization[Title/Abstract] OR Lumen Loss[Title/Abstract] OR LL[Title/Abstract] OR In-segment Late Loss[Title/Abstract] OR Restenosis[Title/Abstract] OR Cardiac Death[Title/Abstract] OR Target Vessel OR Myocardial Infraction[Title/Abstract] OR Adverse Events[Title/Abstract] OR Endpoint[Title/Abstract] OR Endpoints[Title/Abstract]) AND (Human[Filter] OR Randomized Controlled Trial[Filter])

## Web of Science（n=553）

#1: TS=Randomized OR Controlled OR Trial OR Blind

#2: TS=Bioresorbable OR Bioabsorbable

#3: TI=stent OR stents OR Scaffold OR Scaffolds

#4: TS=Target Lesion Failure OR Revascularization OR Lumen Loss OR LL OR In-segment Late Loss OR Restenosis OR Cardiac Death OR Target Vessel Myocardial Infraction OR Adverse Events OR Endpoint OR Endpoints)

#5: TI=Review

#6: TI=Swine OR Pig Or Porcine Or Dog Or Rabbit Or Mouse

#7: #1 AND #2 AND #3 AND #4 NOT (#5 OR #6)

TS=(Randomized OR Controlled OR Trial OR Blind) AND TS=(Bioresorbable OR Bioabsorbable) AND TI=(stent OR stents OR Scaffold OR Scaffolds) AND TS=(Target Lesion Failure OR Revascularization OR Lumen Loss OR LL OR In-segment Late Loss OR Restenosis OR Cardiac Death OR Target Vessel Myocardial Infraction OR Adverse Events OR Endpoint OR Endpoints) NOT (TI=(Review) OR TI=(Swine OR Pig Or Porcine Or Dog Or Rabbit Or Mouse))

## Cochrane Library(n=368)

#1: (stent):ti OR (stents):ti OR (scaffold):ti OR (scaffolds):ti

#2: ("bioresorbable"):ti,ab,kw OR ("bioabsorbable"):ti,ab,kw

#3: (Target Lesion Failure):ti,ab,kw OR (Revascularization):ti,ab,kw OR (Lumen Loss):ti,ab,kw AND (LL):ti,ab,kw AND (In-segment Late Loss):ti,ab,kw

#4: (Restenosis):ti,ab,kw OR (Cardiac Death):ti,ab,kw OR (Target Vessel Myocardial Infraction):ti,ab,kw OR (Adverse Events):ti,ab,kw OR (Endpoint):ti,ab,kw

#5: (endpoints):ti,ab,kw

#6: #1 AND #2 AND (#3 OR #4 OR #5)

## CNKI（n=126）

(SU='支架') AND (SU='可吸收' OR SU=‘可降解’) AND (SU='药物洗脱') NOT (TI='进展' OR TI= 'Meta分析' OR TI=‘综述’)

## Wanfang (n=73)

(主题='支架') AND (主题='药物洗脱') AND (主题='可吸收' OR 主题=‘可降解’) NOT (题名='进展' OR 题名= 'Meta分析' OR 题名=‘综述’)

## VIP (n=101)

(M=支架) AND (M=药物洗脱) AND (M=可吸收 OR M=可降解) NOT (T=进展 OR T= Meta分析 OR T=综述)
